# Relocation of genes generates non-conserved chromosomal segments in *Fusarium graminearum* that show distinct and co-regulated gene expression patterns

**DOI:** 10.1186/1471-2164-15-191

**Published:** 2014-03-13

**Authors:** Chunzhao Zhao, Cees Waalwijk, Pierre JGM de Wit, Dingzhong Tang, Theo van der Lee

**Affiliations:** Plant Research International, Wageningen, The Netherlands; Graduate School Experimental Plant Sciences, Wageningen University, Wageningen, The Netherlands; State Key Laboratory of Plant Cell and Chromosome Engineering, Institute of Genetics and Developmental Biology, Chinese Academy of Sciences, Beijing, China; Graduate University of Chinese Academy of Sciences, Beijing, China; Laboratory of Phytopathology, Wageningen University, Wageningen, The Netherlands

**Keywords:** Gene expression, Non-conserved region, Gene relocation, Secondary metabolite gene cluster, *Fusarium graminearum*

## Abstract

**Background:**

Genome comparisons between closely related species often show non-conserved regions across chromosomes. Some of them are located in specific regions of chromosomes and some are even confined to one or more entire chromosomes. The origin and biological relevance of these non-conserved regions are still largely unknown. Here we used the genome of *Fusarium graminearum* to elucidate the significance of non-conserved regions.

**Results:**

The genome of *F. graminearum* harbours thirteen non-conserved regions dispersed over all of the four chromosomes. Using RNA-Seq data from the mycelium of *F. graminearum*, we found weakly expressed regions on all of the four chromosomes that exactly matched with non-conserved regions. Comparison of gene expression between two different developmental stages (conidia and mycelium) showed that the expression of genes in conserved regions is stable, while gene expression in non-conserved regions is much more influenced by developmental stage. In addition, genes involved in the production of secondary metabolites and secreted proteins are enriched in non-conserved regions, suggesting that these regions could also be important for adaptations to new environments, including adaptation to new hosts. Finally, we found evidence that non-conserved regions are generated by sequestration of genes from multiple locations. Gene relocations may lead to clustering of genes with similar expression patterns or similar biological functions, which was clearly exemplified by the PKS2 gene cluster.

**Conclusions:**

Our results showed that chromosomes can be functionally divided into conserved and non-conserved regions, and both could have specific and distinct roles in genome evolution and regulation of gene expression.

**Electronic supplementary material:**

The online version of this article (doi:10.1186/1471-2164-15-191) contains supplementary material, which is available to authorized users.

## Background

The genus *Fusarium* includes a large group of phytopathogenic fungi, each having a different or partially overlapping host range. *F. graminearum* is an important pathogen on wheat, barley and maize, causing Fusarium head blight [1, 2], while *F. verticillioides* mainly infects maize, causing rot and wilting [3]. In contrast, *F. oxysporum* causes disease on more than 100 different plant species, but specific strains usually infect only a single host species [4]. This has led to the introduction of the *forma specialis* concept in *F. oxysporum,* where strains are categorized according to the host plant they infect, such as tomato or banana [5]. In addition, some strains of *F. oxysporum* may also cause infections in humans [6]. Genomic sequences of several *Fusarium* species have been generated, including *F. graminearum* (strain PH-1), *F. verticillioides* (strain FGSC 7600), *F. pseudograminearum* (strain CS3096), *F. solani* (strain FGSC 9596), *F. fujikuroi* (strain IMI58289) and *F. oxysporum* f. sp. *lycopersici* strain 4287. In addition, 11 different *F. oxysporum* strains, including pathogens from different hosts as well as a biocontrol strain have been sequenced [7–11]. Genome comparisons showed that chromosome XII of *F. fujikuroi* is absent in the genome sequence of related species *F. verticillioides*, while 285 and 820 kb genome sequences at both ends of chromosome IV of *F. verticillioides* are absent in *F. fujikuroi*[11]. Comparing the genome of *F. pseudograminearum* with *F. graminearum* showed that 89.8% of genomic sequence could be aligned at >70% nucleotide identity [7]. Strikingly, *F. oxysporum* 4287 includes 11 core chromosomes and four lineage-specific (LS) chromosomes. LS chromosomes are specific to *F. oxysporum* strain 4287 and have no collinear chromosomes in either *F. graminearum, F. verticillioides* or other *F. oxysporum* strains. The LS chromosomes in *F. oxysporum* 4287 are suggested to have resulted from horizontal transfer from unknown sources [9]. On LS chromosome 14, several effector-encoding genes have been identified that facilitate infection of its host plant [12–14].

In contrast to the high number of chromosomes present in *F. verticillioides* (11), *F. oxysporum* (15) and *F. fujikuroi* (12), *F. graminearum* has only four chromosomes, which probably resulted from fusions of ancestral chromosomes [8]. Comparing the genome of *F. graminearum* isolate PH-1 with *F. graminearum* isolate GZ3639 revealed several regions with high SNP density [8]. In addition, comparison of the genome of *F. graminearum* with the closely related species *F. verticillioides* and *F. oxysporum* revealed several non-conserved regions [9]. Further analysis showed that high SNP density regions match with non-conserved regions. Although many of the genes specifically expressed during plant infection are enriched in non-conserved regions [8], the origin and the biological relevance for these non-conserved regions are still largely unknown. In this study, we explored RNA-Seq data from both conidia and mycelium of *F. graminearum* to investigate the putative effect of gene locations (in conserved or non-conserved regions) on their expression patterns. In addition, by comparing the genome of *F. graminearum* with those of *F. verticillioides* and *F. oxysporum*, we show a possible mechanism for the generation of non-conserved regions.

## Results

Synteny block analysis between the genomes of *Fusarium graminearum*, *F. verticillioides* and *F. oxysporum*.

**Figure 1:**
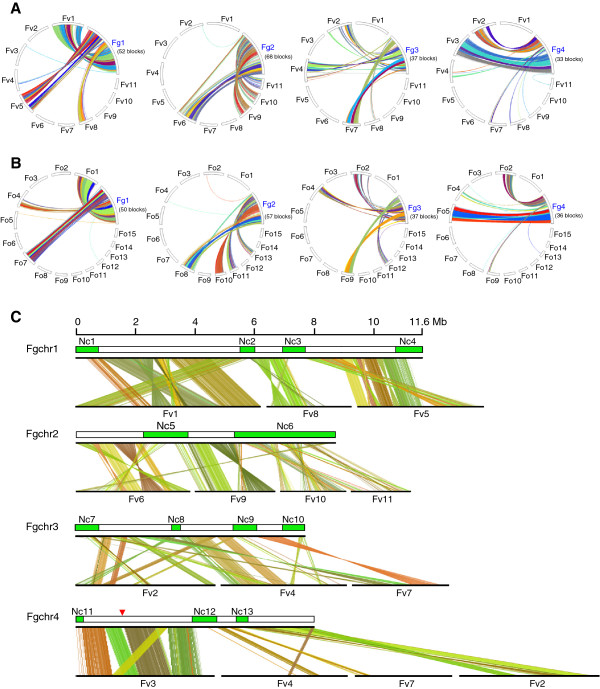
**Comparison of synteny blocks in** ***Fusarium graminearum*** **with** ***F. verticillioides*** **and** ***F. oxysporum*** **.** The genes on each chromosome of *F. graminearum* were compared with the genes of *F. verticillioides* **(A)** and *F. oxysporum* **(B)** and synteny blocks were analyzed by *MCScanX*[46]. The results are shown in a cycle plot. *F. graminearum* contains four chromosomes, *F. verticillioides* 11, and *F. oxysporum* 15, respectively. Each colour represents an independent synteny block, the number of which is shown for each chromosome. **(C)** Detailed analysis of synteny blocks of each chromosome of *F. graminearum* with their collinear chromosomes of *F. verticillioides* by using *MCScanX*. The results are shown as a dual synteny plot. Thirteen non-conserved regions were identified on the chromosomes of *F. graminearum*, which were designated Nc1 to Nc13. Red triangle represents the region of *F. graminearum* that matches unassembled genomic regions of *F. verticillioides* (not shown).

To identify collinear regions on the chromosomes of *F. graminearum* (Fg) and *F. verticillioides* (Fv), we performed synteny block analysis presented in Figure 1. This analysis revealed that *F. graminearum* chromosome 1 (Fgchr 1) is largely collinear with *F. verticillioides* chromosomes (Fvchrs) 1, 5 and 8; Fgchr 2 is collinear with Fvchrs 6, 9, 10 and 11; Fgchr 3 is collinear with Fvchrs 2, 4 and 7; while Fgchr 4 is largely collinear with Fvchrs 2 and 3 (Figure 1A). These major collinear regions between chromosomes indicate that the four *F. graminearum* chromosomes were likely formed primarily through end-to-end joining of two to four smaller ancestral chromosomes, descendants of which still exist in *F. verticillioides*. Similar collinear patterns were observed when comparing the genome of *F. graminearum* with those of *F. oxysporum* (Figure 1B). In addition to these major collinear regions, translocations and inversions of many chromosomal segments are observed in the chromosomes of *F. graminearum*. For instance, the first half of Fgchr 1 is homologous to Fvchr 1, but the order of genomic sequences on Fgchr 1 is different from Fvchr 1. Some chromosomal regions are translocated to new positions, while some are inversed (Figure 1A, C). In addition, thirteen non-conserved regions, which were designated Nc1-Nc13, were found on the four chromosomes of *F. graminearum*. These non-conserved regions exhibit almost no synteny with the chromosomes of *F. verticillioides* (Figure 1C). Notably, although the second half of Fgchr 2 shows collinearity with Fvchrs 10 and 11, a large number of small synteny blocks was identified, indicating multiple rearrangements in this part of chromosome 2 (Figure 1A, C). Similar chromosome rearrangement patterns were observed when the chromosomes of *F. graminearum* were compared with those of *F. oxysporum* 4287 (Figure 1B). We also performed synteny block analysis between *F. oxysporum* and *F. verticillioides*. Consistent with the notion that *F. oxysporum* and *F. verticillioides* are more closely related to each other than to *F. graminearum*, the collinearity between *F. oxysporum* and *F. verticillioides* was much higher (Additional file 1).

### Gene expression analysis on individual chromosomes of *F. graminearum*

**Figure 2:**
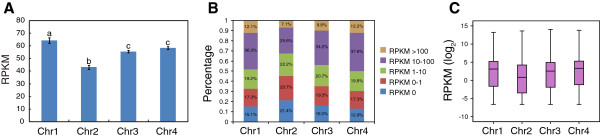
**Evaluation of gene expression levels on the four chromosomes of** ***Fusarium graminearum*** **. (A)** The expression levels of genes on each chromosome were quantified by the number of reads per kilobase per million reads mapped. Genes on chromosome 2 showed relative low expression levels. Different letters represent statistically significant differences (P < 0.01, one-way ANOVA). **(B)** All genes of *F. graminearum* were grouped into five classes according to their RPKM values (0, 0–1, 1–10, 10–100, >100), and the proportion of each class was calculated for each chromosome. **(C)** Box plot analysis of gene expression on each chromosome. The expression of each gene was quantified by its RPKM value. Log2-transformed RPKM values were used to draw a box plot graph for each chromosome.

To investigate a possible correlation between chromosome rearrangements and gene expression patterns on each chromosome of *F. graminearum*, we performed RNA-Seq analysis on mycelium of wild-type isolate PH-1 grown in nutrient-rich medium. Three biologically independent replicates were conducted. In total, 35,103,060 RNA-Seq reads were mapped against the four chromosomes and the expression level of each chromosome was evaluated by using RPKM (reads per kilobase per million mapped reads) values. The averaged expression levels of genes on chromosome 1 are significantly higher than other chromosomes, while the expression levels of genes on chromosome 2 are significantly lower (Figure 2A). Subsequently, we evaluated the expression levels of all predicted genes of *F. graminearum* (Additional file 2) and divided them into five categories based on their RPKM values: RPKM 0, RPKM 0–1, RPKM 1–10, RPKM 10–100 and RPKM >100. The proportion of each category on each chromosome was calculated. As expected, the proportion of weakly expressed genes (e.g. RPKM <1) is higher on Fgchr 2 (45.1%) than on the other chromosomes (30.2-35.3%) (Figure 2B), while the proportion of highly expressed genes (e.g. RPKM >10) on Fgchr 2 (32.7%) is lower than that on the other chromosomes (44-50%). Box plot analysis also showed that relatively more weakly expressed genes (RPKM < 1) are located on Fgchr 2 (*p* value = 4.04e-39, hypergeometric test) (Figure 2C).

### Detailed analysis of chromosome regions with weakly expressed genes in *F. graminearum*

**Figure 3:**
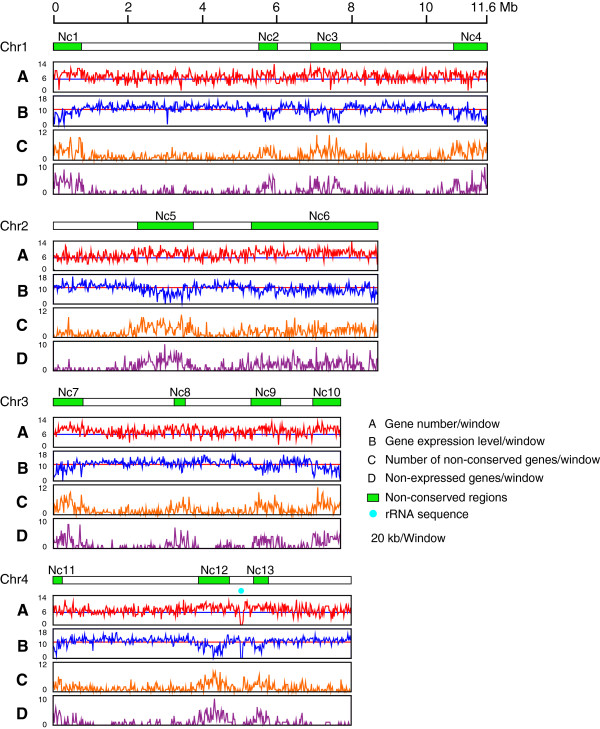
**Gene expression patterns across each of the four chromosomes of** ***Fusarium graminearum*** **.** Each chromosome was divided into 20 kb windows. **(A)** Number of genes per window. The horizontal blue lines represent gene number of 6. **(B)** Gene expression levels per window. Log2-transformed read number per window was used to quantify gene expression levels. Twelve regions with low expression levels were identified on the four chromosomes, which matched non-conserved regions (except Nc8). The horizontal red lines represent an expression level of 10. **(C)** Number of non-conserved genes per window. All genes predicted in *F. graminearum* were matched with the genes of *F. verticillioides* using BLASTn tool and genes without homologs (*p* value > 1E-5) were selected. The number of these non-conserved genes per 20 kb window is shown in each chromosome of *F. graminearum*. **(D)** The number of non-expressed genes in each window of each chromosome is shown. Green boxes represent non-conserved regions in *F. graminearum*. The rDNA region on chromosome 4 is indicated by a blue dot.

To identify positional differences in gene expression, we divided each chromosome into 20 kb windows and for each window the log2-transformed read coverage was used to quantify the expression level. This analysis demonstrated that on Fgchr 2, two large regions, Nc5 (2.37 Mb - 3.82 Mb) and Nc6 (5.44 Mb - 8.89 Mb) exhibited significantly lower expression levels (Lines B in Figure 3) (*p* value < 0.01, Student’s *t*-test) when compared with other parts of the same chromosome or with other chromosomes. In addition, four regions on Fgchr 1, three regions on Fgchr 3 and three regions on Fgchr 4 showed significantly lower gene expression levels. These gene expression patterns were observed for each of three biologically independent replicates (Additional file 3). Subsequently, we determined the number of genes in each of the 20 kb windows. Interestingly the regions with weakly expressed genes showed slightly higher gene density (Lines A in Figure 3). These data demonstrate that the expression of genes is not equally distributed across the chromosomes of *F. graminearum* and that the lower expression is not caused by a lower gene density in these regions.

### Low-expression regions coincide with non-conserved regions

Detailed inspection showed that regions with low levels of expression correspond to non-conserved regions of *F. graminearum* (confer lines B and C in Figure 3). This was further analyzed by comparing genes predicted in the genome of *F. graminearum* to all the genes in *F. verticillioides* using BLASTn (Additional file 4). For 9297 genes in *F. graminearum*, conserved genes were found in *F. verticillioides* (*p* value ≤ 1E-5), while for 4024 genes in *F. graminearum*, no conserved ortholog in *F. verticillioides* was identified (*p* value > 1E-5). When calculating the number of non-conserved genes in each 20 kb window, we found that non-conserved genes are more abundant in chromosomal regions, where genes are weakly expressed (Lines C in Figure 3). This indicates that the regions with weakly expressed genes coincide with the non-conserved regions in *F. graminearum*. In addition, genes that are not expressed in nutrient-rich medium are enriched in non-conserved regions (Lines D in Figure 3).

**Figure 4:**
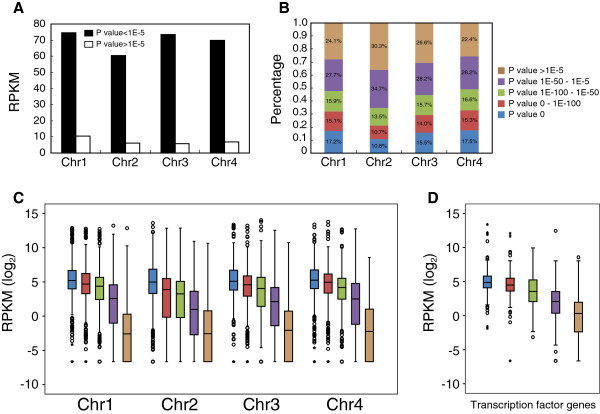
**Gene expression levels in relation to their degree of similarity. (A)** The expression levels of genes with orthologs (*p* value < 1E-5) or genes without orthologs (*p* value > 1E-5) in *F. verticillioides* were quantified by their RPKM values. **(B)** Genes were categorized into five groups according to their degree of similarity with those of *F. verticillioides* (*p* value 0, 0 - 1E-100, 1E-100 - 1E-50, 1E-50 - 1E-5 and >1E-5). The proportion of each category of genes on each chromosome was calculated. **(C)** Box plot analysis of the expression of genes on each chromosome according to their degree of similarity with those of *F. verticillioides*. Log2-transformed RPKM values were used to evaluate gene expression levels. **(D)** Box plot analysis of the expression of 710 transcription factor genes (as identified by Son *et al*., 2011) according to the degree of similarity with those of *F. verticillioides*. Log2-transformed RPKM values were used to evaluate gene expression levels.

To investigate whether non-conserved genes show lower levels of expression, the expression levels of all conserved and non-conserved genes were categorized according to their RPKM values. On each chromosome, the conserved genes showed much higher gene expression levels than non-conserved genes (Figure 4A). Furthermore, we categorized the genes of *F. graminearum* into five groups according to the *p* value of the predicted genes in the BLAST analysis using the predicted genes of *F. verticillioides* as a reference (*p* value 0, 0 - 1E-100, 1E-100 - 1E-50, 1E-50 - 1E-5, >1E-5). Compared to the other three chromosomes, Fgchr 2 contains a higher proportion of non-conserved genes (*p* value > 1E-5) (Figure 4B). In addition, a strong correlation between the degree of gene conservation and gene expression level was observed (Figure 4C), as genes with high similarity to their homologs showed relatively higher expression levels, while genes with less similarity to their homologs showed relatively lower expression levels. To specifically demonstrate this phenomenon the expression levels of all 710 transcription factors, previously identified by Son *et al.*[15] were analyzed. As observed for the expression levels of all genes, the expression levels of genes encoding transcription factors with high similarity to their putative homologs in *F. verticillioides* were higher than genes encoding non-conserved transcription factors (Figure 4D). Furthermore, when each category of genes, grouped by the degree of similarity, was mapped on the four chromosomes of *F. graminearum*, a clear differential gene distribution pattern was observed. Conserved genes tend to cluster together as well as less conserved genes (Additional file 5). Strikingly, no conserved genes (*p* value = 0) are located at one of the terminal regions of Fgchr 3.

### Expression of genes in non-conserved regions is highly variable in different developmental stages

**Figure 5:**
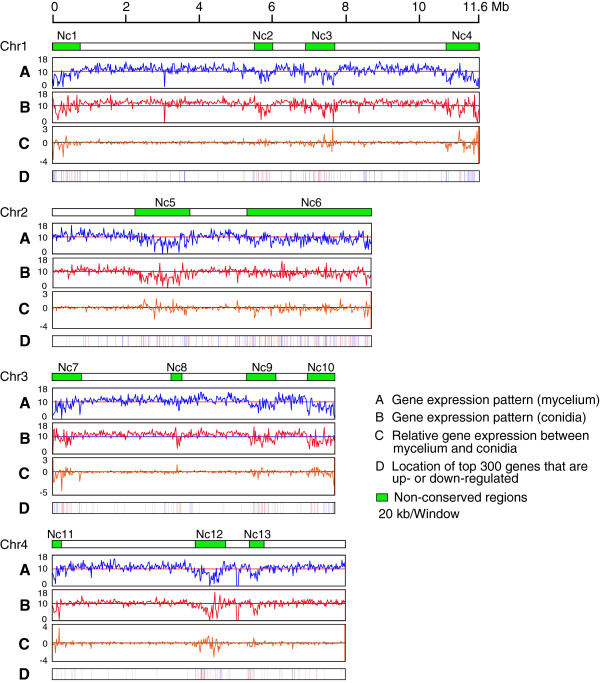
**Comparison of gene expression patterns between mycelium and conidia on the chromosomes of** ***Fusarium graminearum*** **. (A)** Each chromosome was divided into 20 kb windows. Log2-transformed read coverage per window was used to quantify gene expression levels in mycelium of *F. graminearum*. **(B)** Similarly, the Log2-transformed read coverage per window was used to quantify gene expression levels in conidia of *F. graminearum*. **(C)** The ratio of the log2-transformed RPKM values per window between mycelium and conidia. **(D)** The locations of the 300 genes that show the strongest up-regulation comparing mycelium to conidia (red lines) and the 300 genes that show the strongest down-regulated (blue lines) on each chromosome of *F. graminearum*. Green boxes represent non-conserved regions in *F. graminearum*.

To investigate whether gene expression in non-conserved regions is invariably low, or whether it is related to a specific growth stage, RNA-Seq analysis was performed on conidia of *F. graminearum* isolate PH-1 produced in mung bean medium. Three biologically independent replicates were analyzed and in total 40,615,406 reads were obtained. Gene expression patterns on each chromosome of *F. graminearum* were analyzed with the obtained RNA-Seq data. Firstly, similar gene expression patterns on each chromosome of *F. graminearum* were observed for the three biological replicates (Additional file 6). Secondly, also in conidia nearly all non-conserved regions exhibited significantly lower gene expression levels than conserved regions (*p* value < 0.01, Student’s *t*-test). However, some parts of non-conserved regions, especially in regions Nc4 and Nc7 showed expression levels similar to conserved regions (lines B in Figure 5). In addition, we compared the gene expression levels between conidia and mycelium in each 20 kb window on each chromosome. Strikingly, the relative expression (e.g. expression in mycelium/expression in conidia) of genes located in conserved regions is relatively constant, while the relative expression of genes in non-conserved regions is sometimes highly variable (lines C in Figure 5). Furthermore, we selected the 300 genes that exhibit the strongest up- and down-regulation between the two different developmental stages and mapped their locations on each chromosome of *F. graminearum*. We found that these highly induced or repressed genes are significantly abundant in non-conserved regions (*p* value = 2.57e-49, hypergeometric test) (lines D in Figure 5). These data suggest that gene expression in non-conserved regions might be important for *F. graminearum* to respond to external stimuli or during specific phases of its life cycle, while gene expression in conserved regions are more stable and are more likely to be involved in housekeeping functions.

### Non-conserved regions are enriched for genes encoding secreted proteins and enzymes required for the production of secondary metabolites

**Figure 6:**
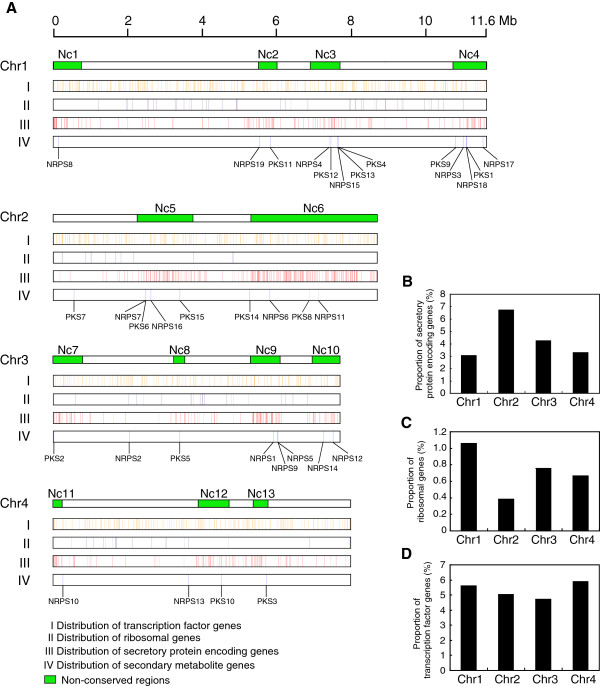
**Distribution of different categories of genes on each chromosome of** ***F. graminearum*** **. (A)** 710 transcription factor genes, 547 genes encoding secreted proteins, 98 ribosomal genes and 34 genes encoding enzymes putatively involved in the production of secondary metabolites are mapped on the chromosomes. Genes encoding secreted proteins and genes encoding enzymes for production of secondary metabolites are enriched in weakly expressed regions, ribosomal genes are predominantly found in conserved regions, while transcription factor genes are randomly distributed. **(B)** Proportion of genes encoding secreted proteins on each chromosome of *F. graminearum*. **(C)** Proportion of ribosomal protein genes on each chromosome of *F. graminearum*. **(D)** Proportion of transcription factor encoding genes on each chromosome of *F. graminearum*. Green boxes represent non-conserved regions identified in *F. graminearum*.

To further understand the biological relevance of non-conserved regions, we examined which categories of genes are enriched in the non-conserved regions. Firstly, we studied the distribution of all genes encoding secreted proteins in *F. graminearum*. Previously, Brown *et al*. [16] identified 574 genes encoding secreted proteins in the *F. graminearum* genome [16]. Distribution of these genes on each chromosome of *F. graminearum* showed that 384 locate in non-conserved regions and 190 in conserved regions (Figure 6A), suggesting that they are significantly enriched in non-conserved regions (*p* value = 4.32e-69, hypergeometric test). Moreover, a higher proportion of genes encoding secreted proteins were observed on chromosome 2 (*p* value = 5.77e-15, hypergeometric test) (Figure 6B). Secondly, the distribution of secondary metabolite genes, including 15 polyketide synthase (PKS) genes and 19 non-ribosomal peptide (NRPS) genes [17] also appeared to be unevenly distributed: 14 PKS genes and 17 NRPS genes locate in non-conserved regions, while only one PKS gene and two NRPS genes were found in conserved regions. This indicates that genes involved in secondary metabolism are also enriched in non-conserved regions. (*p* value = 3.09e-9, hypergeometric test). In contrast, ribosomal genes that are essential for growth are enriched in conserved regions (*p* value = 2.56e-14, hypergeometric test) and fewer ribosomal genes are identified on chromosome 2 of *F. graminearum* (Figure 6A, C). In contrast, transcription factor-encoding genes were randomly distributed on each chromosome of *F. graminearum* (Figure 6A, D) (*p* value = 0.03, hypergeometric test).

### Non-conserved regions are enriched for relocated genes

**Figure 7:**
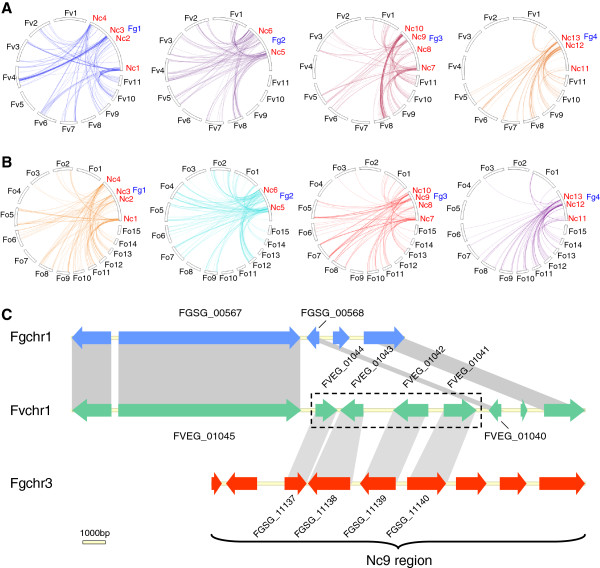
**Frequent gene relocations in non-conserved regions of the four chromosomes of** ***Fusarium graminearum*** **. (A-B)** Genes matching their orthologs on non-collinear chromosomes of *F. verticillioides* **(A)** or *F. oxysporum* **(B)** are present on each of the four chromosomes of *F. graminearum*. **(C)** One representative example showing relocation of four genes to region Nc9. Orthologs of four *F. verticillioides* genes (FVEG_01044, FVEG_01043, FVEG_01042 and FVEG_01041) were not identified in the collinear chromosomal region, but were found in region Nc9 of *F. graminearum*.

As mentioned above, 9297 genes in *F. graminearum* have homologs in *F. verticillioides*. To further demonstrate that genes with their putative homologs in *F. verticillioides* are likely to be true orthologs, the homologs in *F. verticillioides* were aligned against the genes in *F. graminearum* using BLASTn and we found that 9208 gene pairs are the reciprocal best blast hits, suggesting that they are orthologs (Additional file 4). These genes can be further divided into two groups based on their collinearity with their orthologs. Most *F. graminearum* genes have their putative orthologs on one of the collinear chromosomes of *F. verticillioides*, while some genes have their putative orthologs located on non-collinear chromosomes of *F. verticillioides*. The number of genes matching to putative orthologs on each chromosome of *F. verticillioides* is presented in Table 1. For example, on Fgchr 1, 2794 genes have a putative ortholog on the collinear chromosomes (1, 5, 8) of *F. verticillioides,* whereas for 187 genes, putative orthologs were shown on non-collinear chromosomes of *F. verticillioides*. We then mapped all these genes on the chromosomes of *F. graminearum* and found that non-conserved regions are highly enriched for these genes (Figure 7A). This indicates that non-conserved regions in *F. graminearum* are enriched in genes that have undergone relocation. A similar pattern was observed when comparing *F. graminearum* with *F. oxysporum* (Figure 7B). Further evidence for these relocations comes from the fact that in 355 (80.5%) of these relocations a single gene was relocated, but in 46 (10.4%) cases two neighbouring genes and in 40 (9.1%) cases three or more neighbouring genes were relocated together. Figure 7C shows one example of a relocation of four neighbouring genes in *F. graminearum*. On Fvchr1 four genes were found between genes FVEG_01045 and FVEG_01040, whereas no genes were identified between orthologs FGSG_00567 and FGSG_00568 in *F. graminearum*, indicating that these four genes have been relocated to a new location in *F. graminearum*. BLAST analysis of the four *F. verticillioides* genes showed that their orthologs in *F. graminearum* are relocated to the non-conserved region Nc9 of Fgchr 3.

**Table 1:** **Number of genes on each chromosome of** ***Fusarium graminearum*** **that have orthologs on each chromosome of** ***F. verticillioides***

	Fvchr1	Fvchr2	Fvchr3	Fvchr4	Fvchr5	Fvchr6	Fvchr7	Fvchr8	Fvchr9	Fvchr10	Fvchr11
Fgchr1	**1478**	15	24	74	**958**	17	13	**358**	12	19	13
Fgchr2	31	26	17	35	17	**772**	12	27	**568**	**333**	**281**
Fgchr3	24	**485**	14	**681**	18	7	**623**	72	17	15	20
Fgchr4	8	**632**	**1034**	**152**	8	11	103	68	20	14	18

Values in bold type are the numbers of genes that have orthologs on collinear chromosomes of *F. verticillioides*, while values in regular type are the numbers of genes that have orthologs on non-collinear chromosomes of *F. verticillioides*.

To understand whether genes that are relocated to non-conserved regions are also affected in expression, we compared the expression levels of relocated genes with non-relocated genes. This analysis indicated that expression of relocated genes was significantly lower than non-relocated genes under the conditions examined (confer A and B in Additional file 7).

### Secondary metabolite gene clusters are assembled in non-conserved regions by gene relocations

**Figure 8:**
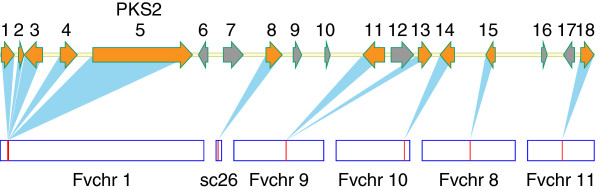
**PKS2 gene cluster is assembled in a non-conserved region by gene relocations.** The *PKS2* gene and its flanking genes are matched with their orthologs in *F. verticillioides*. The numbers represent the genes of *F. graminearum* that are listed in Table 2. Orange colour represents genes that have orthologs, while grey colour indicates genes that have no clear orthologs in *F. verticillioides*. The boxes below represent the chromosomes of *F. verticillioides* and also the locations of orthologs on each chromosome. Sc26 is an unmapped supercontig in *F. verticillioides*.

As shown above, most of secondary metabolite genes are located in non-conserved regions, and previous studies have shown that genes producing secondary metabolites are often clustered [17], but whether these secondary metabolite gene clusters are assembled by gene relocations is still unknown. Here we found that gene clusters, such as *PKS1*, *PKS2*, *PKS6*, *PKS8* and *PKS10*, are probably formed by gene relocations, as genes in these clusters have orthologs that are located on different chromosomes of *F. verticillioides*. This is exemplified by the PKS2 gene cluster of which most genes have high similarity to their orthologs (Table 2). The ortholog of PKS2 gene is located on chromosome 1 of *F. verticillioides*. Other genes that are typically associated with secondary metabolite gene clusters, such as MFS monosaccharide transporter, NAD binding oxidoreductase, alcohol dehydrogenase, cytochrome P450 family protein and C6 transcription factor, have their orthologs located on the chromosomes 8, 9, 10 and 11 of *F. verticillioides*, respectively (Figure 8 and Table 2). This result suggests that secondary metabolite gene clusters could be formed by gene relocations as was found for other genes in the non-conserved regions.

**Table 2:** **Analysis of PKS2 secondary metabolite gene cluster**

Num	Gene ID	Putative function	Orthologs in ***Fusarium verticillioides***		
			Gene ID	Chromosome	***p*** value
1	FGSG_12582	Sterol 3-beta-glucosyltransferase	FVEG_00073	Fvchr 1	0
2	FGSG_12583	Putative bile acid 7-alpha protein	FVEG_00076	Fvchr 1	5.51E-155
3	FGSG_04692	Acyltransferase	FVEG_00077	Fvchr 1	0
4	FGSG_04693	Integral membrane protein PTH11	FVEG_00078	Fvchr 1	0
5	FGSG_04694 (PKS2)	Polyketide synthase	FVEG_00079	Fvchr 1	0
6	FGSG_04695	Hypothetical protein	-	-	-
7	FGSG_04696	Hypothetical protein	-	-	-
8	FGSG_04697	Hypothetical protein	FVEG_13993	Sc26	0
9	FGSG_04698	Hypothetical protein	-	-	-
10	FGSG_04699	Hypothetical protein	-	-	-
11	FGSG_04700	MFS monosaccharide transporter	FVEG_10988	Fvchr 9	9.16E-83
12	FGSG_04701	ARCA-like protein	-	-	-
13	FGSG_04702	NAD binding oxidoreductase	FVEG_10989	Fvchr 9	1.58E-55
14	FGSG_04703	Alcohol dehydrogenase	FVEG_13775	Fvchr 10	7.36E-61
15	FGSG_04704	Cytochrome P450 family protein	FVEG_07686	Fvchr 8	0
16	FGSG_04705	RTA1 like protein	-	-	-
17	FGSG_04706	RTA1 domain-containing protein	-	-	-
18	FGSG_12584	C6 transcription factor	FVEG_10599	Fvchr 11	6.25E-52

“-” represents that no ortholog was identified in *F. verticillioides*.

### The origin of lineage-specific chromosomes in *F. oxysporum*

*F. oxysporum* f. sp. *lycopercisi* isolate 4287 contains eleven core chromosomes and four lineage-specific (LS) chromosomes [9]. Comparison of core chromosomes of *F. oxysporum* with those of *F. verticillioides* through synteny analysis revealed several non-conserved regions (Additional file 1), especially at the end of chromosomes 1 and 2. To determine whether the four LS chromosomes and non-conserved regions on core chromosomes of *F. oxysporum* are enriched for gene relocations, we selected all genes of *F. oxysporum* with homologs on a non-collinear chromosome of *F. verticillioides* and assigned these genes to each chromosome of *F. oxysporum*. The four LS chromosomes appear to be enriched for genes that have relocated from core chromosomes (Additional file 8). Gene relocations were also supported by the observation that multiple groups of adjacent genes on LS chromosomes showed collinearity with their homologs on the chromosomes of *F. verticillioides* (Additional file 9). In addition, relocation of genes was also observed in non-conserved regions of core chromosomes of *F. oxysporum*, especially in one telomere proximal region of chromosomes 1 and 2. In addition, synteny block analysis of chromosomes in *F. oxysporum* was performed and we found that most of the genes present on LS chromosomes 3 and 6 are duplicated, which is consistent with previous findings [9]. Some genes on LS chromosome 14 might originate from LS chromosomes 3 or 6. Interestingly, genes on LS chromosome 15 are duplicated from core chromosome 1 (Additional file 10), suggesting that LS chromosome 15 originally arose from core chromosome.

## Discussion

Genome comparison between closely related species may help us to understand the mechanism of genome evolution and apprehend how species evolve to adapt to new environments [18–20]. Comparing the genome of *Homo sapiens* with the closely related species chimpanzee showed that 98.76% of the genomic sequences are similar [21], but 1,576 putative inversions were identified on the chromosomes of *H. sapiens*[22] that are likely to be involved in genome evolution. Large-scale translocations and inversions appear to have also occurred in the ascomycete *Saccharomyces cerevisiae*[23, 24]. Here, we compared the genome of *F. graminearum* with that of two closely related species *F. verticillioides* and *F. oxysporum*. Again, a large number of translocations and inversions were identified. Next to these translocations and inversions of large chromosomal segments, non-conserved regions are commonly discovered in closely related species. For instance, synteny analysis of the genome of *Aspergillus nidulans* with related species *A. fumigatus* and *A. oryzae* showed that around 78% of the genome could be mapped to conserved syntenic blocks, while the remaining genomic sequences lack significant syntenic blocks [25]. In addition, syntenic analysis of 12 sequenced *Drosophila* species showed that on average 66% of each genome was covered by syntenic blocks [26], indicating that the location of the remaining 34% of the genome is not conserved. Comparing the genome of *F. graminearum* with those of *F. verticillioides* and *F. oxysporum*, thirteen non-conserved regions were identified. The presence of these non-conserved regions suggests that genome evolution has occurred unevenly across the chromosomes. Although non-conserved regions frequently occur in the genomes of many species, their origin and biological relevance are still largely unknown.

The development of RNA-Seq technology [27] allowed us to evaluate the global gene expression patterns along chromosomes [28, 29]. In this study, we used RNA-Seq data obtained from the mycelium of the sequenced isolate PH-1 of *F. graminearum* grown in nutrient-rich medium to investigate the gene expression pattern along whole chromosomes. Interestingly, we found two striking features in *F. graminearum*: (i) there is a strong correlation between the degree of gene conservation and gene expression level and (ii) genes in the non-conserved regions showed lower expression levels than genes in the conserved regions. In addition, comparing gene expression levels between conidia and mycelium, we found that the expression of genes in non-conserved regions is highly variable, while the expressions of genes in conserved regions is surprisingly stable. This indicates the expression of genes in conserved regions is less dependent on fungal development (e.g. in conidia *vs.* mycelium), while the expression of genes in non-conserved regions seems more developmentally regulated. Furthermore, house-keeping genes are more abundant in conserved regions, while genes required in specific developmental stages or environmental conditions are found more often in non-conserved regions. This conclusion was supported by the fact that genes encoding secreted proteins or involved in the production of secondary metabolites, which are induced under specific conditions [8, 30–32], are more abundant in non-conserved regions, while ribosomal genes are mainly located in conserved regions.

Gene enrichment in specific chromosomal regions has also been studied in other species. For instance, secondary metabolite genes are enriched in subtelomeric regions of *Aspergillus* species. The rapid rearrangement of subtelomeric regions may promote the rapid evolution of these genes to become species-specific attributes [25, 33]. In the plant pathogenic fungus *Verticillium dahliae*, *in planta*-expressed genes are enriched in lineage-specific genomic regions that have developed by extensive chromosomal reshuffling, which was suggested to drive evolution of virulence [34]. In *F. oxysporum*, genes encoding secreted effectors and virulence factors are more abundant in LS chromosomes, while few house-keeping genes are identified on LS chromosomes [9]. The drivers and biological relevance of gene enrichment in specific chromosome regions are still not fully understood. One possible reason could be that clustering of genes with similar expression patterns may facilitate co-regulation of specific sets of genes under specific conditions or developmental stages. This phenomenon has been observed in many species, such as yeast, mouse and human [35–38]. Secondly, changes in gene expression levels have also been shown to be important in adaptive evolution [39, 40], so perhaps enrichment of genes in non-conserved regions could facilitate them to rapidly change their expression pattern.

Our data indicate that genes in non-conserved regions are weakly expressed, but how the expression of these genes is suppressed is still unknown. Previous studies have shown that gene position in the nucleus is associated with their transcriptional regulation, for instance, the nuclear periphery was considered as a zone for transcriptionally repressed genes [41]. This type of organization could also occur in *F. graminearum* and non-conserved regions could form specific sub-compartments of the nucleus with repressed gene expression.

The non-conserved regions occur widely, but how they are generated is still unclear. In this study, many gene relocations were identified in non-conserved regions of *F. graminearum*, in contrast to conserved regions. Gene relocations have been described previously in *S. cerevisiae* where the *DAL* gene cluster, including six genes, was formed by gene relocations from six different loci [42]. In *F. graminearum* and *F. sporotrichioides*, trichothecene biosynthesis requires genes at three loci: the 12-gene *TRI* cluster, a second locus with two genes (*TR1* and *TRI16*), and a third locus with one gene (*TRI101*). However, in the more distantly related species *F. equiseti*, both *TRI1* and *TRI101* are located within the *TRI* core cluster suggesting that the latter two genes have been relocated during evolution of *F. equiseti*[43]. We also showed that four successive genes were relocated to a non-conserved region in *F. graminearum*. However, how these genes are marked for relocation is still obscure. Based on our findings that genes with low similarity to their homologs have low expression levels (refer to Figure 4C), we hypothesize that the relocated genes in non-conserved chromosome regions already had a low expression level before they were relocated. Possibly, in the three dimensional organization of chromosomes in the interphase these weakly expressed genes are in close proximity with the likewise weakly expressed non-conserved regions. Such a close proximity might facilitate targeted relocation of weakly expressed genes to one of these non-conserved regions.

In *F. oxysporum*, four LS chromosomes and 11 core chromosomes were identified. It was suggested that these four LS chromosomes were generated by horizontal transfer from a yet unknown fungal source [9]. Also for the dispensable chromosomes in *Zymoseptoria tritici* horizontal gene transfer was hypothesized [44]. Although horizontal gene transfer is one of the reasonable explanations for the origin of LS chromosomes in *F. oxysporum*[9], other mechanisms or events could also contribute to the formation of LS regions. In this study, the frequent gene relocations identified in non-conserved regions of *F. graminearum* drove us to hypothesize that the generation of LS chromosomes in *F. oxysporum* could also have included extensive gene relocations. This hypothesis was supported by the fact that around 700 genes on LS chromosomes have a homolog in *F. verticillioides*, and in 22 cases, two or more adjacent genes are collinear with their homologs. In addition, large part of LS chromosome 15 represents a duplication of the telomere proximal region of core chromosome 1, suggesting that it might originate from the core chromosome. Based on the studies by us and others [9], we proposed that most likely horizontal gene transfer from other fungal species and gene relocations within the species both occurred in *F. oxysporum*.

## Conclusions

Taken together, our data demonstrate that chromosomes of *F. graminearum* show distinct conserved and non-conserved regions. Subsequent gene expression analysis showed that genes in these regions exhibit different expression patterns. Genes showing high and stable expression levels are more abundant in conserved regions, while genes that are induced or repressed in specific developmental stages or under different environmental conditions (mung bean medium versus liquid CM) are significantly abundant in non-conserved regions. This type of genome arrangement may not only facilitate the co-regulation of specific sets of genes, but could also enable fungi to maintain on the one hand the required conservation of house-keeping genes and on the other to accelerate the evolution of species-specific genes to rapidly adapt to new environments or new hosts. Moreover, due to the selective transcription of genes in non-conserved regions, this could prevent organisms spending too much energy in transcription and translation of evolving genes that do not have acquired full functionality yet.

## Methods

### RNA isolation and RNA-Seq

*Fusarium graminearum* wild-type isolate PH-1 was used in this study. To prepare conidia and mycelium for RNA isolation, PH-1 was grown in 400 ml liquid mung bean medium for 3 days to produce conidia (25°C, 200 rpm). The conidia were collected by centrifugation. 10e5 conidia of PH-1 were transferred to 400 ml liquid complete medium and subsequently incubated for 30 h to produce mycelium (25°C, 200 rpm). Mycelium was harvested from liquid CM medium by filtration and grounded in liquid nitrogen using a mortar and pestle. The conidia and mycelium were used for RNA extraction using TRIzol reagent (Invitrogen, Cat. No. 15596–018) according to manufacturer’s instructions. The quality of RNA was analyzed by Agilent 2100. RNA-Seq was performed according to protocols described previously [45].

### Analysis of gene homology

Gene homology was evaluated in CLC genomic workbench. The gene database of *F. graminearum*, *F. verticillioides* and *F. oxysporum* were downloaded from Broad Fusarium Comparative Database. The gene database (fasta file format) of each *Fusarium* species were imported into CLC, and “BLASTn” option was used to align the genes of *F. graminearum* against the genes of *F. verticillioides* or *F. oxysporum* using default settings. Genes of *F. graminearum* were grouped according to their *p* value after aligning with the genes of *F. verticillioides* by using BLASTn.

The identification of orthologs between *Fusarium* species was based on two criteria: (i) a cutoff of *p* value of 1E-5, and (ii) reciprocal best blast hits. For the genes in conserved chromosomal regions, synteny block analysis was also explored for the definition of orthologous relationship.

### Synteny block analysis

Synteny block analysis was performed according to the program *MCScanX* with a small modification [46]. The gene data sets (fasta format file) of *F. graminearum*, *F. verticillioides* and *F. oxysporum* were downloaded from the Broad *Fusarium* database. The local blast database pools of *F. verticillioides* and *F. oxysporum* were created by using program formatdb. All genes in *F. graminearum* were analyzed against the gene database pool of *F. verticillioides* and *F. oxysporum*, respectively, by using BLAST tool. The BLAST results were exported in m8 format. Besides, gff file containing the information of the chromosome number (e.g. Fg1), gene name (e.g. FGSG_00001), start position and stop position of each gene on each chromosome of *Fusarium* species was prepared. The BLAST file and gff file were imported for synteny block analysis according to the procedure described in the manual of *MCScanX*. The criteria for the synteny analysis are as follow: match score 50, match size > 5, gap_penalty −1, overlap_window 5, max gaps 25. Finally, two types of output (dual synteny plot and circle plot) were obtained by using two downstream programs.

### Analysis of relocated genes

According to the BLASTn result, all *F. graminearum* genes with high similarity (*p* value ≤ 1E-5) in *F. verticillioides* or *F. oxysporum* were collected, from which we selected genes that have their putative orthologs on non-collinear chromosomes of *F. verticillioides* or *F. oxysporum*. The map of these genes to their orthologs was performed by using *MCScanX* software [46]. Furthermore, the sequences of all these putative orthologs from either *F. verticillioides* or *F. oxysporum* were collected and matched with all predicated genes in *F. graminearum* using BLASTn tool to identify the best hits to show that they are the bidirectional best hits.

### Gene expression analysis

To evaluate gene expression along chromosomes, RNA-Seq reads were mapped to chromosome sequences of *F. graminearum* using software available in the CLC Genomics Workbench. The RNA-Seq reads were mapped to each chromosome by using “RNA-Seq analysis” option with default settings. The number of reads matched to each chromosome was calculated and subsequently the expression level of each chromosome was evaluated by using RPKM (reads per kilobase per million mapped reads) values. Similarly, to evaluate the expression of each gene, the transcript database of *F. graminearum* were imported in CLC and the expression level of each gene was evaluated by RPKM value.

To draw the gene expression level along each chromosome, we divided the chromosomes into portions of 20 kb. The read coverage of each 20 kb window was calculated and log2-transformed reads coverage in each window was used to compare gene expression levels. To compare the gene expression level between conidia and mycelium, reads coverage of each window was compared and log2-transformed reads coverage fold change was used to evaluate gene expression differences. The total gene number and the number of non-conserved genes in each 20 kb window were calculated manually based on the criteria described above.

To analyze the expression levels of conserved and non-conserved genes, the transcript sequences of conserved and non-conserved genes were collected and assembled, respectively. Also in this case the PRKM value was used to evaluate the expression levels of conserved and non-conserved genes. Box plot analysis of gene expression was performed by using SPSS software.

### Availability of supporting data

The data sets supporting the results of this article are included within the article and its additional files. Six Illumina sequence data used in this study are available in the NCBI GEO repository (accession number GSE55477, http://www.ncbi.nlm.nih.gov/geo/query/acc.cgi?acc=GSE55477).

## Electronic supplementary material

Additional file 1: **Synteny block analysis of** ***Fusarium oxysporum*** **with the genomic sequence of** ***F. verticillioides*** **.** The genomic sequence of *F. oxysporum* strain 4287 was used to compare the genomic sequence of *F. verticillioides* by using program *MCScanX*. Eleven core chromosomes of *F. oxysporum* contain collinear chromosomes in *F. verticillioides*, while four LS chromosomes do not contain collinear chromosomes in *F. verticillioides*. Red triangles represent non-conserved regions identified on the chromosome 1 and 2 of *F. oxysporum*. (PDF 764 KB)

Additional file 2: **Genome-wide gene expression analysis.** Excel file showing the expression of all predicted genes in both conidia and mycelium of *Fusarium graminearum* (three biological replicates each). (XLS 2 MB)

Additional file 3: **Gene expression pattern analysis using RNA-Seq data obtained from mycelium of** ***F. graminearum*** **.** Each chromosome of *F. graminearum* was divided into 20 kb windows. For each window, the log2-transformed reads coverage was drawn to show gene expression patterns along each chromosome of *F. graminearum*. The gene expression patterns analyzed by three biologically independent RNA-Seq data obtained from mycelium of *F. graminearum* are shown. Green boxes represent non-conserved regions identified in *F. graminearum*. (PDF 146 KB)

Additional file 4: **Putative orthologs of** ***Fusarium graminearum*** **genes in** ***F. verticillioides*** **and** ***F. oxysporum*** **.** All predicted genes of *F. graminearum* were matched to their orthologs in both *F. verticillioides* and *F. oxysporum* by using BLASTn. (XLS 2 MB)

Additional file 5: **Distribution of genes on each chromosome of** ***F. graminearum*** **according to their similarity to genes in** ***F. verticillioides*** **.** Genes on each chromosome were divided into five groups according to their degree of similarity. Genes with a low degree of similarity to their orthologs are enriched in weakly expressed regions, while genes with a high degree of similarity to their orthologs are enriched in highly expressed regions. Green boxes represent non-conserved regions identified in *F. graminearum*. Note the absence of conserved genes (*p* value=0) at the telomeric region of Chr3, corresponding to Nc10. (PDF 182 KB)

Additional file 6: **Gene expression pattern analysis using RNA-Seq data obtained from conidia of** ***F. graminearum*** **.** Each chromosome of *F. graminearum* was divided into 20 kb windows. For each window, the log2-transformed reads coverage was drawn to show gene expression patterns along each chromosome of *F. graminearum*. The gene expression patterns analyzed by three biologically independent RNA-Seq data obtained from the conidia of *F. graminearum* are shown. Green boxes represent non-conserved regions identified in *F. graminearum*. (PDF 144 KB)

Additional file 7: **Comparison of the expression of relocated and non-relocated genes.** All genes that have orthologs in *F. verticillioides* were divided into two groups: genes that are relocated to non-conserved regions and genes that are not relocated. Box plot analysis shows that the expression levels of relocated genes in non-conserved regions **(B)** are lower than non-relocated genes **(A)**. Asterisk indicates significant difference (*p* value<0.01, Student’s *t*-test). (PDF 41 KB)

Additional file 8: **LS chromosomes in** ***F. oxysporum*** **are enriched for gene relocations.** Genes that match their orthologs on non-collinear chromosomes of *F. verticillioides* are distributed on the chromosomes of *F. oxysporum*. Four LS chromosomes, Fo3, Fo6, Fo14 and Fo15, show multiple gene relocations. In addition, the telomere proximal regions of the core chromosomes, especially chromosome 1 and 2, show multiple gene relocations. (PDF 420 KB)

Additional file 9: **Group of neighboring genes on LS chromosomes of** ***Fusarium oxysporum*** **that are collinear with their homologs in** ***F. verticillioides*** **.** (DOC 98 KB)

Additional file 10: **Synteny block analysis of each chromosome against other chromosomes in** ***F. oxysporum*** **.** The genomic sequence of each chromosome was used to compare other chromosomes by using program *MCScanX*. Genomic sequence duplications were identified between four LS chromosomes. Remarkably, LS chromosome 15 is duplicated from the telomere proximal region of core chromosome 1. (PDF 338 KB)

## Abbreviations

Nc: Non-conserved
RPKM: Reads per kilobase per million mapped reads
LS: Lineage-specific
SNP: Single nucleotide polymorphism.
