# Supplementary figures and images for: Relocation of genes generates non-conserved chromosomal segments in Fusarium graminearum that show distinct and co-regulated gene expression patterns
Source: BMC Genomics. 2014 Mar 13;15(1):191. doi: 10.1186/1471-2164-15-191 (PMC4022177; doi:10.1186/1471-2164-15-191)

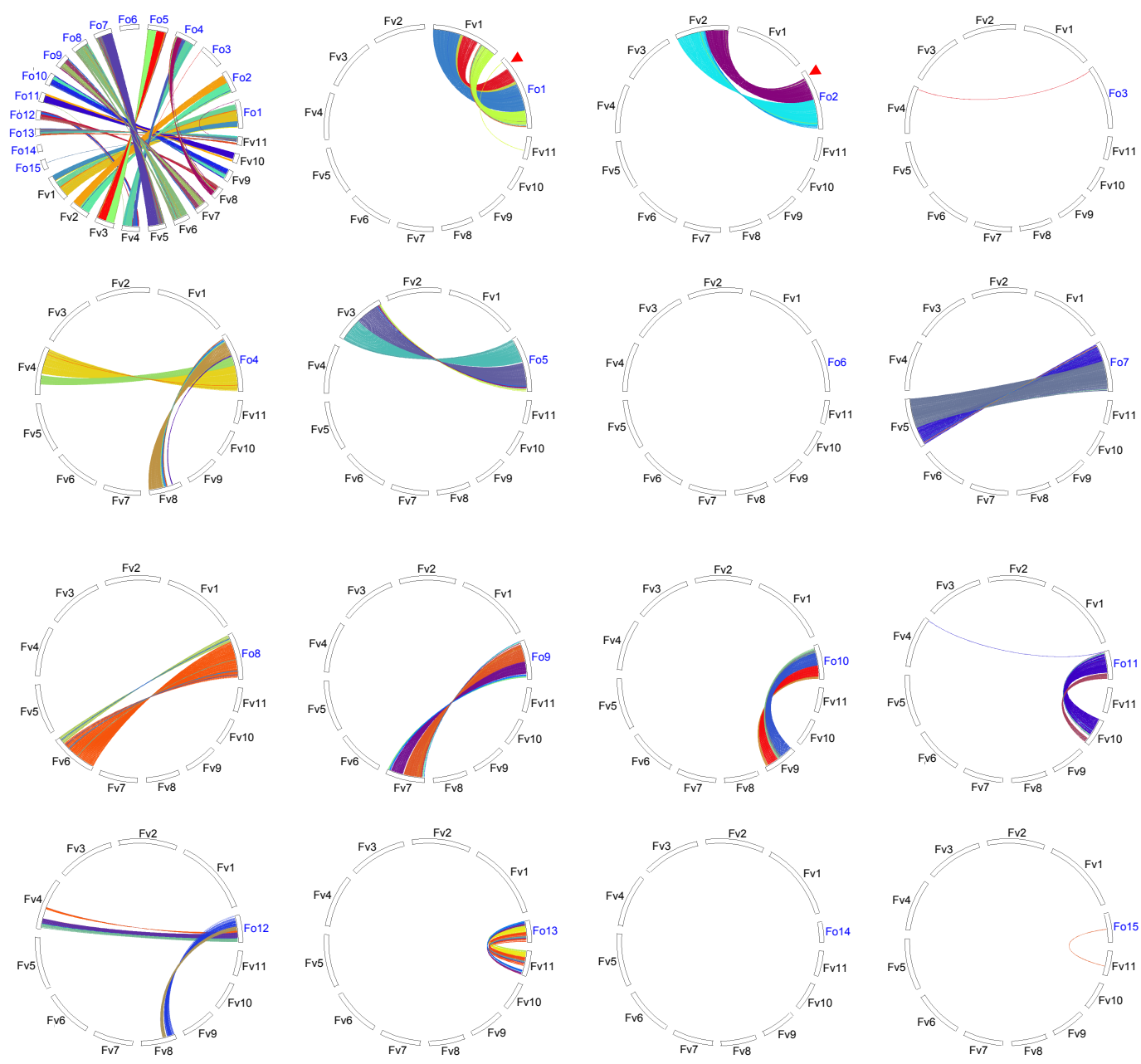

Supplement: Supplementary file 1 — Additional file 1: Synteny block analysis of Fusarium oxysporum with the genomic sequence of F. verticillioides . The genomic sequence of F. oxysporum strain 4287 was used to compare the genomic sequence of F. verticillioides by using program MCScanX. Eleven core chromosomes of F. oxysporum contain collinear chromosomes in F. verticillioides, while four LS chromosomes do not contain collinear chromosomes in F. verticillioides. Red triangles represent non-conserved regions identified on the chromosome 1 and 2 of F. oxysporum. (PDF 764 KB) [file 12864_2013_7029_MOESM1_ESM.pdf]

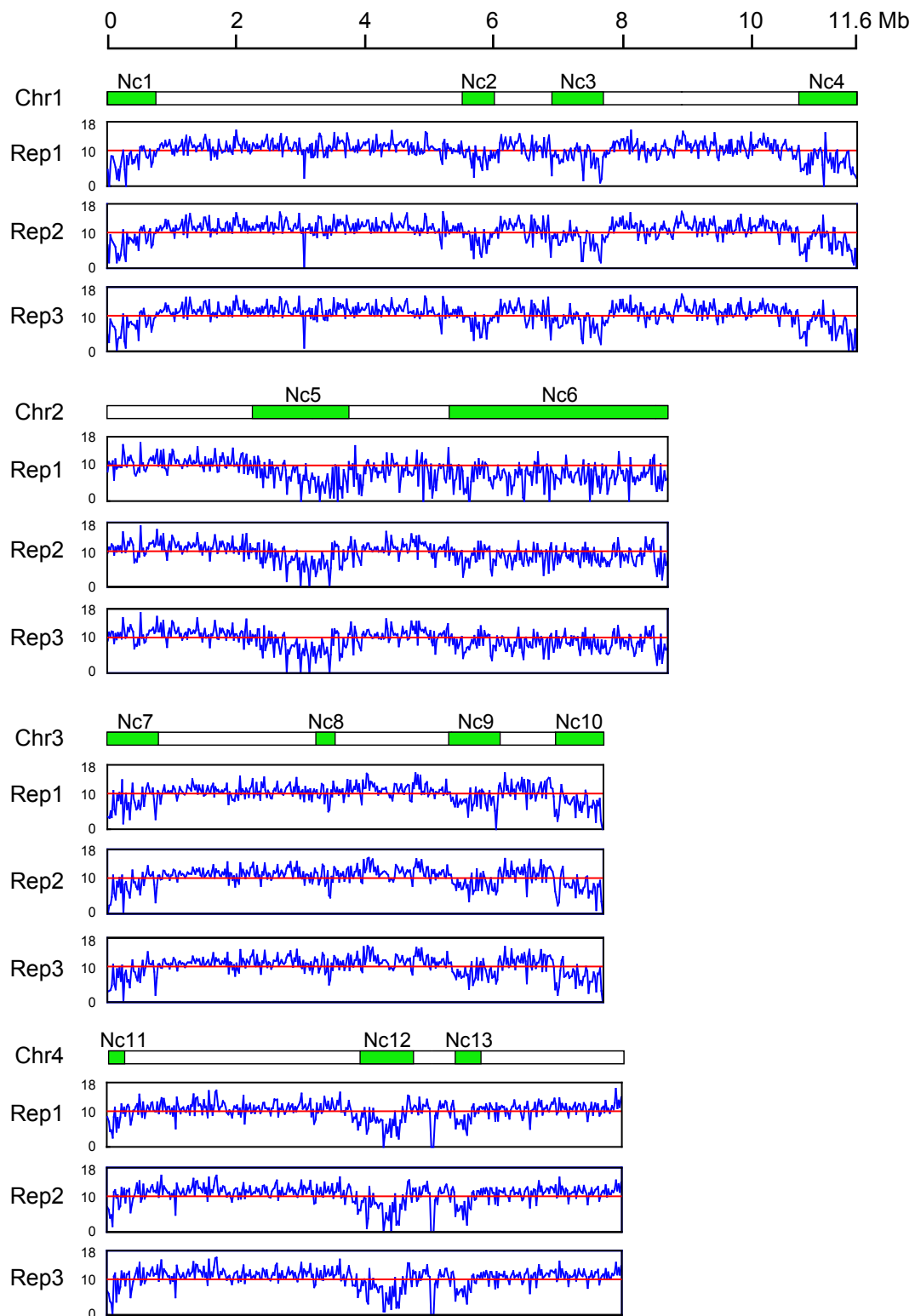

Supplement: Supplementary file 3 — Additional file 3: Gene expression pattern analysis using RNA-Seq data obtained from mycelium of F. graminearum . Each chromosome of F. graminearum was divided into 20 kb windows. For each window, the log2-transformed reads coverage was drawn to show gene expression patterns along each chromosome of F. graminearum. The gene expression patterns analyzed by three biologically independent RNA-Seq data obtained from mycelium of F. graminearum are shown. Green boxes represent non-conserved regions identified in F. graminearum. (PDF 146 KB) [file 12864_2013_7029_MOESM3_ESM.pdf]

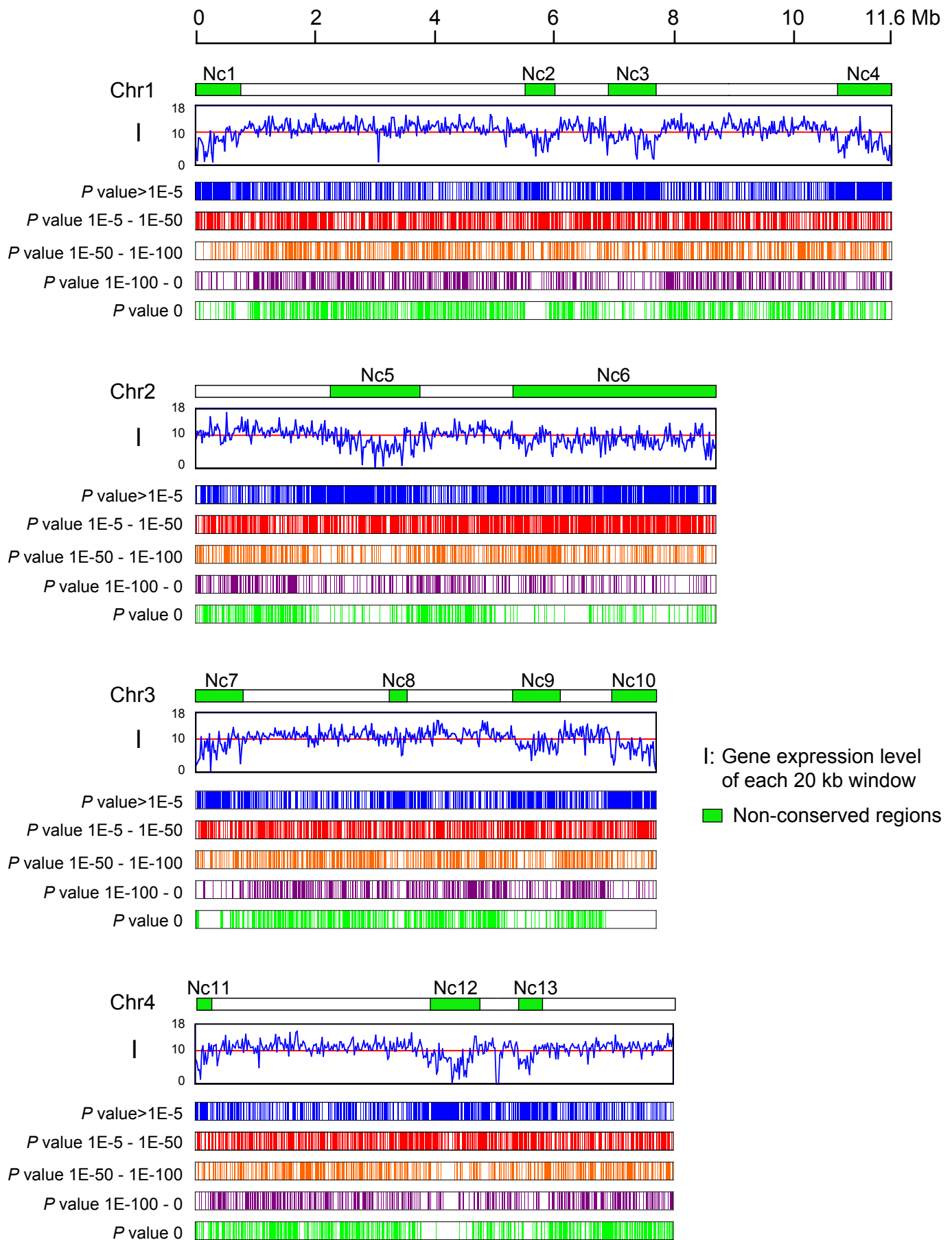

Supplement: Supplementary file 5 — Additional file 5: Distribution of genes on each chromosome of F. graminearum according to their similarity to genes in F. verticillioides . Genes on each chromosome were divided into five groups according to their degree of similarity. Genes with a low degree of similarity to their orthologs are enriched in weakly expressed regions, while genes with a high degree of similarity to their orthologs are enriched in highly expressed regions. Green boxes represent non-conserved regions identified in F. graminearum. Note the absence of conserved genes (p value=0) at the telomeric region of Chr3, corresponding to Nc10. (PDF 182 KB) [file 12864_2013_7029_MOESM5_ESM.pdf]

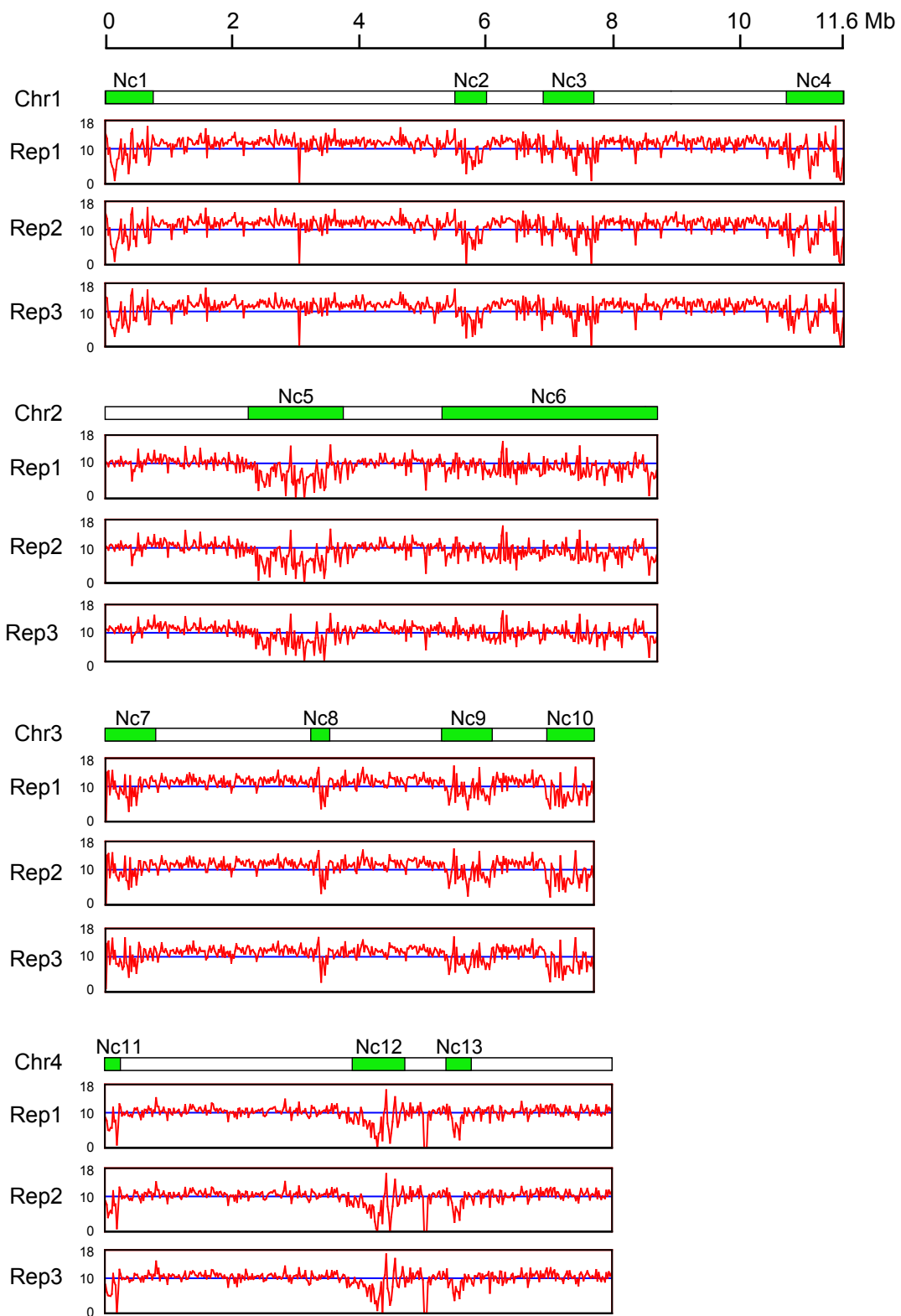

Supplement: Supplementary file 6 — Additional file 6: Gene expression pattern analysis using RNA-Seq data obtained from conidia of F. graminearum . Each chromosome of F. graminearum was divided into 20 kb windows. For each window, the log2-transformed reads coverage was drawn to show gene expression patterns along each chromosome of F. graminearum. The gene expression patterns analyzed by three biologically independent RNA-Seq data obtained from the conidia of F. graminearum are shown. Green boxes represent non-conserved regions identified in F. graminearum. (PDF 144 KB) [file 12864_2013_7029_MOESM6_ESM.pdf]

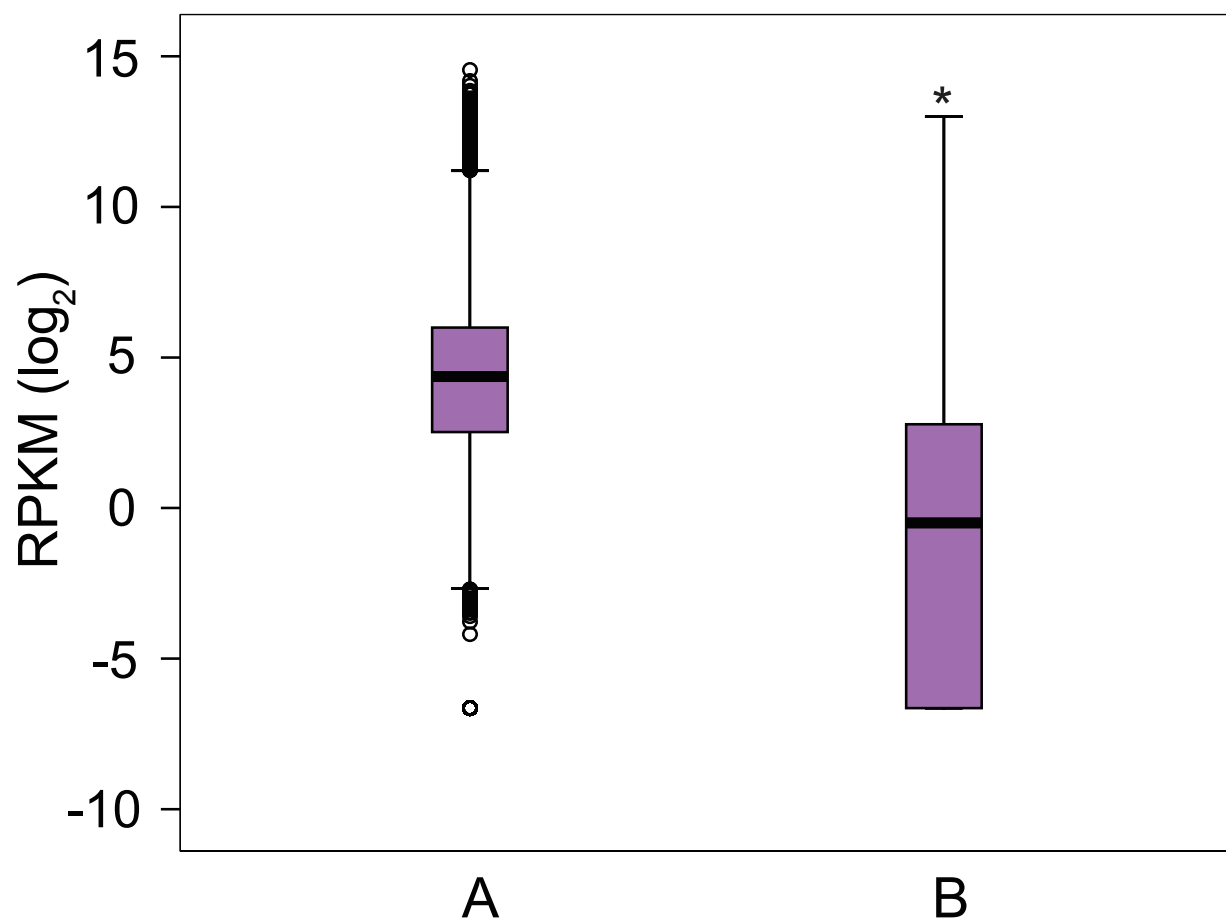

Supplement: Supplementary file 7 — Additional file 7: Comparison of the expression of relocated and non-relocated genes. All genes that have orthologs in F. verticillioides were divided into two groups: genes that are relocated to non-conserved regions and genes that are not relocated. Box plot analysis shows that the expression levels of relocated genes in non-conserved regions (B) are lower than non-relocated genes (A). Asterisk indicates significant difference (p value<0.01, Student’s t-test). (PDF 41 KB) [file 12864_2013_7029_MOESM7_ESM.pdf]

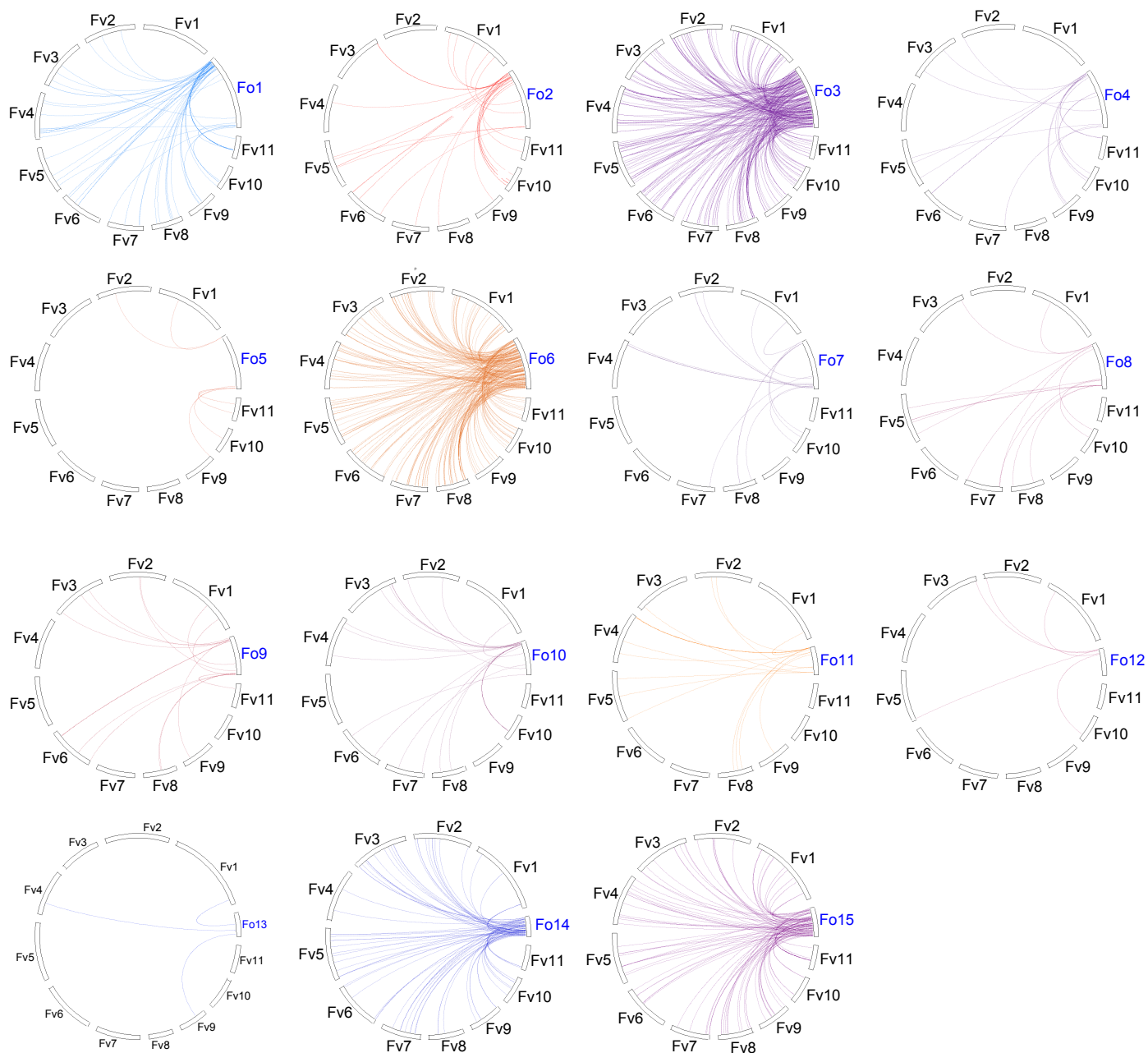

Supplement: Supplementary file 8 — Additional file 8: LS chromosomes in F. oxysporum are enriched for gene relocations. Genes that match their orthologs on non-collinear chromosomes of F. verticillioides are distributed on the chromosomes of F. oxysporum. Four LS chromosomes, Fo3, Fo6, Fo14 and Fo15, show multiple gene relocations. In addition, the telomere proximal regions of the core chromosomes, especially chromosome 1 and 2, show multiple gene relocations. (PDF 420 KB) [file 12864_2013_7029_MOESM8_ESM.pdf]

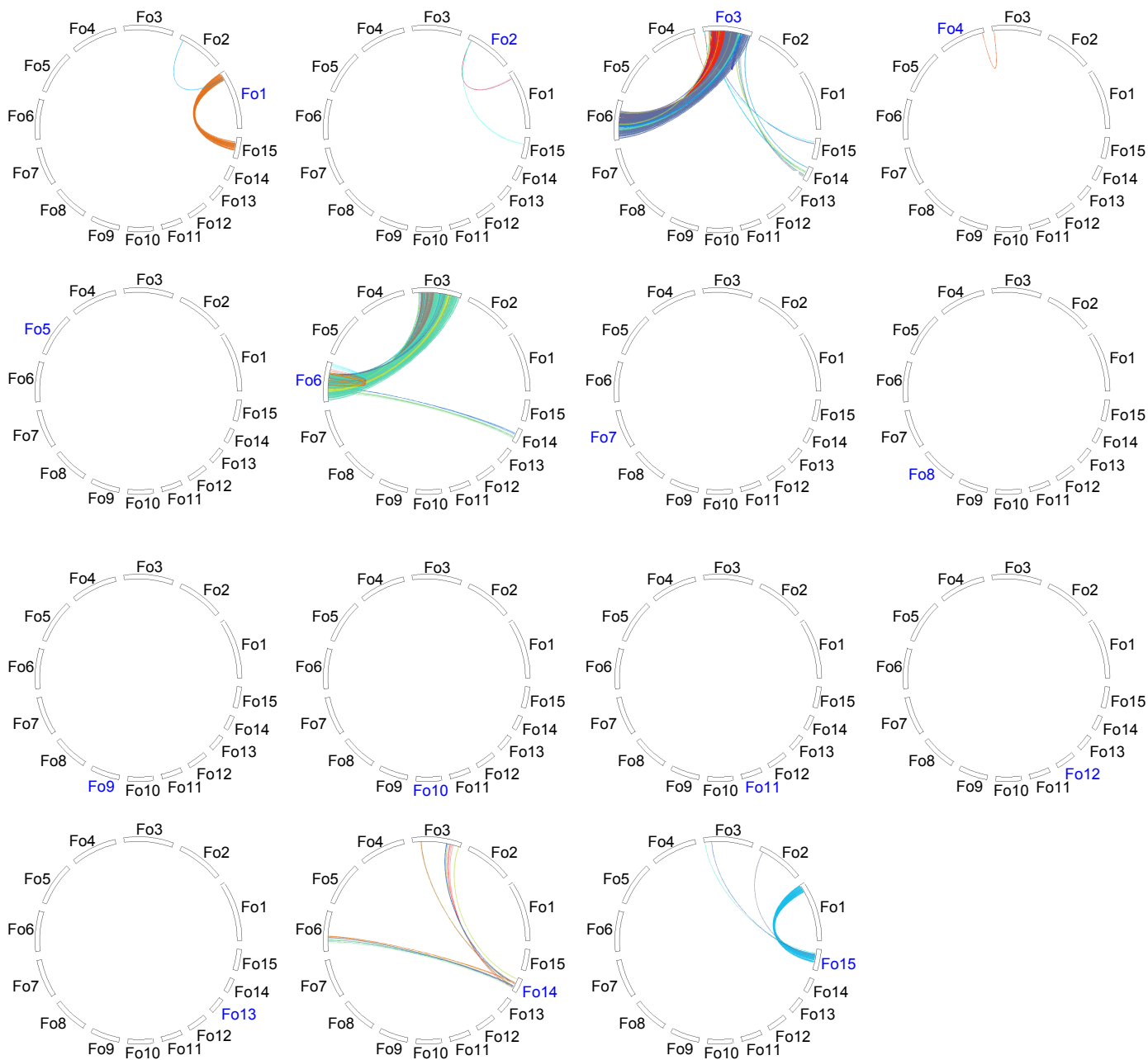

Supplement: Supplementary file 10 — Additional file 10: Synteny block analysis of each chromosome against other chromosomes in F. oxysporum . The genomic sequence of each chromosome was used to compare other chromosomes by using program MCScanX. Genomic sequence duplications were identified between four LS chromosomes. Remarkably, LS chromosome 15 is duplicated from the telomere proximal region of core chromosome 1. (PDF 338 KB) [file 12864_2013_7029_MOESM10_ESM.pdf]
