# Supplementary material for: Relocation of genes generates non-conserved chromosomal segments in Fusarium graminearum that show distinct and co-regulated gene expression patterns
Source: BMC Genomics. 2014 Mar 13;15(1):191. doi: 10.1186/1471-2164-15-191 (PMC4022177; doi:10.1186/1471-2164-15-191)
Supplement: Supplementary file 9 — Additional file 9: Group of neighboring genes on LS chromosomes of Fusarium oxysporum that are collinear with their homologs in F. verticillioides . (DOC 98 KB) [file 12864_2013_7029_MOESM9_ESM.doc]

Additional file 9 Group of neighboring genes on LS chromosomes of *Fusarium oxysporum* that are collinear with their homologs in *F. verticillioides*.

| Group | Gene ID | Chromosome | Homologs in *Fusarium verticillioides* | | |
| --- | --- | --- | --- | --- | --- |
|  |  |  | GeneID | Chromosome | *p* value |
| 1 | FOXG_12459 | Fochr3 | FVEG_06980 | Fvchr7 | 8E-09 |
|  | FOXG_12461 | Fochr3 | FVEG_06981 | Fvchr7 | 1E-08 |
| 2 | FOXG_12413 | Fochr3 | FVEG_08664 | Fvchr10 | 2E-21 |
|  | FOXG_12412 | Fochr3 | FVEG_08665 | Fvchr10 | 2E-65 |
| 3 | FOXG_14888 | Fochr3 | FVEG_10773 | Fvchr11 | 6E-16 |
|  | FOXG_14887 | Fochr3 | FVEG_10774 | Fvchr11 | 6E-16 |
| 4 | FOXG_06503 | Fochr3 | FVEG_13461 | Fvchr8 | 0 |
|  | FOXG_06502 | Fochr3 | FVEG_13462 | Fvchr8 | 3E-14 |
|  | FOXG_06501 | Fochr3 | FVEG_13464 | Fvchr8 | 9E-56 |
|  | FOXG_06500 | Fochr3 | FVEG_13465 | Fvchr8 | 1E-12 |
| 5 | FOXG_06759 | Fochr3 | FVEG_13461 | Fvchr8 | 0 |
|  | FOXG_06760 | Fochr3 | FVEG_13462 | Fvchr8 | 4E-47 |
|  | FOXG_06761 | Fochr3 | FVEG_13464 | Fvchr8 | 9E-56 |
|  | FOXG_06762 | Fochr3 | FVEG_13465 | Fvchr8 | 1E-12 |
| 6 | FOXG_06866 | Fochr3 | FVEG_13465 | Fvchr8 | 0 |
|  | FOXG_06870 | Fochr3 | FVEG_13464 | Fvchr8 | 2E-48 |
|  | FOXG_06871 | Fochr3 | FVEG_13462 | Fvchr8 | 4E-36 |
| 7 | FOXG_16139 | Fochr3 | FVEG_13462 | Fvchr8 | 4E-36 |
|  | FOXG_16140 | Fochr3 | FVEG_13464 | Fvchr8 | 2E-33 |
|  | FOXG_16144 | Fochr3 | FVEG_13465 | Fvchr8 | 0 |
| 8 | FOXG_07112 | Fochr6 | FVEG_02865 | Fvchr5 | 8E-71 |
|  | FOXG_07100 | Fochr6 | FVEG_02866 | Fvchr5 | 9E-07 |
| 9 | FOXG_06967 | Fochr6 | FVEG_05732 | Fvchr3 | 5E-18 |
|  | FOXG_06971 | Fochr6 | FVEG_05733 | Fvchr3 | 9E-12 |
|  | FOXG_06970 | Fochr6 | FVEG_05734 | Fvchr3 | 2E-21 |
|  | FOXG_06969 | Fochr6 | FVEG_05737 | Fvchr3 | 8E-09 |
| 10 | FOXG_14078 | Fochr6 | FVEG_06980 | Fvchr7 | 8E-09 |
|  | FOXG_14076 | Fochr6 | FVEG_06981 | Fvchr7 | 1E-08 |
| 11 | FOXG_14124 | Fochr6 | FVEG_08664 | Fvchr10 | 2E-21 |
|  | FOXG_14125 | Fochr6 | FVEG_08665 | Fvchr10 | 1E-60 |
| 12 | FOXG_06957 | Fochr6 | FVEG_13464 | Fvchr8 | 3E-36 |
|  | FOXG_06958 | Fochr6 | FVEG_13462 | Fvchr8 | 2E-49 |
|  | FOXG_06959 | Fochr6 | FVEG_13461 | Fvchr8 | 0 |
| 13 | FOXG_07172 | Fochr6 | FVEG_13461 | Fvchr8 | 0 |
|  | FOXG_07173 | Fochr6 | FVEG_13462 | Fvchr8 | 1E-51 |
|  | FOXG_07174 | Fochr6 | FVEG_13464 | Fvchr8 | 2E-50 |
|  | FOXG_07175 | Fochr6 | FVEG_13465 | Fvchr8 | 1E-12 |
| 14 | FOXG_07321 | Fochr6 | FVEG_13461 | Fvchr8 | 0 |
|  | FOXG_07322 | Fochr6 | FVEG_13462 | Fvchr8 | 4E-47 |
|  | FOXG_07323 | Fochr6 | FVEG_13464 | Fvchr8 | 2E-53 |
|  | FOXG_07324 | Fochr6 | FVEG_13465 | Fvchr8 | 1E-12 |
| 15 | FOXG_16235 | Fochr6 | FVEG_13465 | Fvchr8 | 0 |
|  | FOXG_16239 | Fochr6 | FVEG_13464 | Fvchr8 | 2E-33 |
|  | FOXG_16240 | Fochr6 | FVEG_13462 | Fvchr8 | 4E-36 |
| 16 | FOXG_14290 | Fochr14 | FVEG_02567 | Fvchr6 | 0 |
|  | FOXG_14289 | Fochr14 | FVEG_02568 | Fvchr6 | 0 |
| 17 | FOXG_16453 | Fochr14 | FVEG_05746 | Fvchr3 | 1E-19 |
|  | FOXG_16452 | Fochr14 | FVEG_05747 | Fvchr3 | 9E-16 |
| 18 | FOXG_14382 | Fochr15 | FVEG_10080 | Fvchr9 | 6E-72 |
|  | FOXG_14384 | Fochr15 | FVEG_10081 | Fvchr9 | 4E-47 |
| 19 | FOXG_14360 | Fochr15 | FVEG_10478 | Fvchr11 | 0 |
|  | FOXG_14359 | Fochr15 | FVEG_10479 | Fvchr11 | 0 |
|  | FOXG_14358 | Fochr15 | FVEG_10480 | Fvchr11 | 0 |
|  | FOXG_14357 | Fochr15 | FVEG_10482 | Fvchr11 | 0 |
|  | FOXG_14356 | Fochr15 | FVEG_10483 | Fvchr11 | 0 |
|  | FOXG_14355 | Fochr15 | FVEG_10484 | Fvchr11 | 0 |
|  | FOXG_14354 | Fochr15 | FVEG_10485 | Fvchr11 | 0 |
|  | FOXG_14352 | Fochr15 | FVEG_10486 | Fvchr11 | 6E-116 |
|  | FOXG_14351 | Fochr15 | FVEG_10487 | Fvchr11 | 0 |
| 20 | FOXG_16778 | Fochr15 | FVEG_11801 | Fvchr7 | 1E-94 |
|  | FOXG_16781 | Fochr15 | FVEG_11802 | Fvchr7 | 2E-07 |
| 21 | FOXG_14336 | Fochr15 | FVEG_12257 | Fvchr4 | 3E-42 |
|  | FOXG_14335 | Fochr15 | FVEG_12258 | Fvchr4 | 1E-57 |
| 22 | FOXG_14327 | Fochr15 | FVEG_13769 | Fvchr10 | 0 |
|  | FOXG_14330 | Fochr15 | FVEG_13770 | Fvchr10 | 1E-08 |
